# Prenatal exposure to metal mixtures and lung function in children from the New Hampshire birth cohort study

**DOI:** 10.1016/j.envres.2023.117234

**Published:** 2023-10-02

**Authors:** Antonio J. Signes-Pastor, Leyre Notario-Barandiaran, Margaret Guill, Juliette Madan, Emily Baker, Brian Jackson, Margaret R. Karagas

**Affiliations:** a Department of Epidemiology, Geisel School of Medicine, Dartmouth College, NH, USA; b Unidad de Epidemiología de la Nutrición. Universidad Miguel Hernández, Alicante, Spain; c CIBER de Epidemiología y Salud Pública (CIBERESP), Instituto de Salud Carlos III (ISCIII), Madrid, Spain; d Instituto de Investigación Sanitaria y Biomédica de Alicante (ISABIAL), Spain; e Department of Pediatrics, Dartmouth College, Lebanon, NH, USA; f Department of Obstetrics & Gynecology, Division of Maternal Fetal Medicine, Dartmouth-Hitchcock Medical Center, Lebanon, NH, USA; g Department of Biological Sciences, Dartmouth College, Hanover, NH, USA

**Keywords:** Mixture, Toxic metals, Toxic metalloids, Trace elements, children’s environmental health, Respiratory outcomes, Biomarkers of exposure

## Abstract

Prenatal exposure to metals/metalloids, even at common US population levels, may pose risks to fetal health, and affect children’s lung function. Yet, the combined effects of simultaneous prenatal exposures on children’s lung function remain largely unexplored. This study analyzed 11 metals (As speciation, Cd, Co, Cu, Mo, Ni, Pb, Sb, Se, Sn, Zn) in maternal urine during weeks 24–28 of gestation and evaluated lung function, including forced vital capacity (FVC) and forced expiratory volume in the first second of expiration (FEV_1_), in 316 US mother-child pairs at around age 7. We used Bayesian Kernel Machine Regression (BKMR), weighted quantile sum regression (WQSR), and multiple linear regression to examine the association between metal mixture exposure and children’s lung function, adjusting for maternal smoking, child age, sex, and height. In BKMR models assessing combined exposure effects, limited evidence of metal non-linearity or interactions was found. Nevertheless, Co, As species, and Pb showed a negative association, while Mo exhibited a positive association with children’s FVC and FEV_1_, with other metals held constant at their medians. The weighted index, from WQSR analysis assessing the cumulative impact of all metals, highlighted prenatal Mo with the highest positive weight, and Co, As, and Sb with the most substantial negative weights on children’s FVC and FEV_1_. Urinary Co and Pb were negatively associated with FVC (β = −0.09, 95% confidence interval (CI) (−0.18; −0.01) and β = −0.07, 95% CI (−0.13; 0.00), respectively). Co was also negatively associated with FEV_1_ (β = −0.09, 95% CI (−0.18; 0.00). There was a negative association between As and FVC, and a positive association between Mo and both FVC and FEV_1_, though with wide confidence intervals. Our findings suggest that prenatal trace element exposures may impact children’s lung function, emphasizing the importance of reducing toxic exposures and maintaining adequate nutrient levels.

## 1. Introduction

Prenatal exposures to environmental chemical, such as metals and metalloids (hereafter, referred to as “metals”) are of concern due to their potential to cross the placental barrier and accumulate in fetal tissues (Li et al., 2019; Ruan et al., 2022). Gestation represents a sensitive period when even low levels of toxic exposures may impact the fetal development and cause long-term health effects (Dou et al., 2022; Liu et al., 2018; Ma et al., 2023; Ruan et al., 2022). Pregnant women are concurrently exposed to a myriad of contaminants including potential toxic metals as well as essential, nutrient elements, which could result in synergistic, additive, or antagonistic effects (Bhagat et al., 2021; Henn et al., 2014). However, the current literature is largely comprised of single element studies among highly exposed populations, which may underestimate the possible multicollinearity and complex interactions of exposure mixtures (Hernández et al., 2020; Heys et al., 2016).

Previous studies have reported an association between individual toxic metal exposures and lung function in children (Little et al., 2017; Madrigal et al., 2018, 2021; Pan et al., 2020; Wu et al., 2019), especially among highly exposed populations (Recio-Vega et al., 2015; Zeng et al., 2017). In the United States (US), a cross-sectional analysis of the National Health and Nutrition Examination Survey (NHANES) found that exposure to metals individually, including lead, cadmium, and mercury were negatively associated with pulmonary function among 6–19 years old (Feng et al., 2022). A study conducted on children and adolescents with asthma between the ages of 6 and 17 in Illinois found that urine manganese and lead concentrations were inversely associated with forced expiratory volume in 1 s (FEV_1_), forced vital capacity (FVC) and mid-exhalation forced expiratory flow rate (FEF_25–75%_) (Madrigal et al., 2018). These studies have focused on the individual assessment of toxic metals, leaving aside essential metals and the effect of metal mixtures, particularly among child populations.

Few studies have investigated children’s respiratory effects of prenatal metal exposures (Ahmed et al., 2017; McRae et al., 2022; Rahman et al., 2011; Rosa et al., 2022; Signes-Pastor et al., 2021). In prior work in our US pregnancy cohort, we found that prenatal arsenic exposure was associated with lower children’s lung function, as measured with spirometry at ~7 years of age (Signes-Pastor et al., 2021), as well as an increased risk of respiratory symptoms and infections during the first year of life (Farzan et al., 2016). Drinking water contaminated with arsenic at around the time of birth was also found to be associated with later mortality risk from bronchiectasis in an ecologic analysis from Chile (Smith et al., 2006). In Mexico, children aged 4–7 years with higher prenatal exposure to lead had an increased risk of wheeze (McRae et al., 2022). Despite some studies in adult population suggested a potential role of metal mixtures exposure on lung development (Sobel et al., 2022; Wu et al., 2022), we lack a clear understanding of the impact of prenatal exposure to metal mixtures on children’s lung function (Bobb et al., 2018; Carrico et al., 2015).

Therefore, we aimed to investigate the association between urine concentrations of a mixture of 11 metals in maternal urine samples collected during pregnancy (i.e., arsenic (As speciation), cadmium (Cd), cobalt (Co), copper (Cu), molybdenum (Mo), nickel (Ni), lead (Pb), antimony (Sb), selenium (Se), tin (Sn), and zinc (Zn) in relation to 7-year-old children’s spirometry measurements of lung capacity and function.

## 2. Methods

### 2.1. Study population

Our research involved the inclusion of mother-child pairs who participated in the New Hampshire Birth Cohort Study (NHBCS), a longitudinal study with the objective of examining how environmental factors impact the health of both mothers and children. The NHBCS actively recruited pregnant women between the ages of 18 and 45, during the gestational period of approximately 24–28 weeks, mainly from prenatal clinics located in rural areas of New Hampshire, where the predominant racial group is white. To be eligible for participation, individuals were required to meet certain criteria, including English literacy, having a private and unregulated water system at their residence (such as a private well), no plans to relocate during pregnancy, and the expectation of a single birth. The study received approval from the Committee for the Protection of Human Subjects at Dartmouth College, and all participants provided written informed consent in accordance with ethical guidelines. To avoid self-plagiarism, please refer to prior studies for additional information on the study population and methodology, including laboratory analysis and spirometry measurements briefly described below (Signes-Pastor et al., 2021, 2022).

### 2.2. Sample collection

At approximately 24–28 weeks into gestation, mothers were asked to provide a urine sample using polyethylene sterile containers. The collected samples were processed and frozen at a temperature of −80 °C within 24 h, in preparation for subsequent analysis (Signes-Pastor et al., 2021, 2022).

### 2.3. Laboratory analysis

The concentrations of metals in maternal urine were analyzed at the Trace Element Analysis Core located at Dartmouth College. To measure the concentrations of Cd, Co, Cu, Mo, Ni, Pb, Sb, Se, Sn, and Zn in urine, an Agilent 8900 inductively coupled plasma-mass spectrometry (ICP-MS) system operating in direct solution acquisition mode was utilized. Measurement of urinary As requires As speciation to remove unmetabolized species such as arsenobetaine which are not considered toxic. Therefore, the analysis of urinary As species concentrations involved the use of the Agilent 8900 ICP-MS coupled with an Agilent liquid chromatograph 1260 equipped with a Thermo AS7 column measuring 2 × 250 mm, along with a Thermo AG7 guard column measuring 2 × 50 mm (Jackson, 2015; Signes-Pastor et al., 2020). Additionally, the measurement of urinary specific gravity was conducted using an automatic temperature-compensating handheld refractometer (PAL-10S; ATAGO Co Ltd).

The limit of detection (LOD) was determined by calculating the mean of the blank concentrations, adding three times their standard deviation (SD), and multiplying the result by the dilution factor. The LOD values for each metal of interest in different analysis batches can be found in Table S1. Information regarding the percentage of values below the LOD and imputed values is also presented in Table S1. The imputation of the $\text{LOD}/\sqrt{2}$ value occurred only when the ICP-MS standard calibration curve provided zero or negative values (Lubin et al., 2004). However, the remaining urine concentrations below the LOD were not imputed, taking advantage of the ICP-MS wide linear dynamic range (EFSA, 2009). Each analysis batch included multiple blanks and certified reference material samples (e.g., NIST 2669 level I and level II). The recoveries of the certified reference material were approximately 100%.

### 2.4. Lung function

At approximately 7 years of age, children underwent spirometry to evaluate their lung function, along with assessments of their age, height, and weight. Spirometry is a reliable method for detecting respiratory abnormalities (Okyere et al., 2021; Zhang et al., 2022). Trained personnel conducted the spirometry tests in a single session, following the guidelines established by the American Thoracic Society and European Respiratory Society (Beydon et al., 2007; Crapo et al., 1995). These tests were overseen by a pediatric pulmonologist (**MG**), and the staff received comprehensive training to ensure accurate and high-quality performance. Prior to the tests, each child received personalized education and pre-testing to optimize their performance. The spirometry tests were conducted without the use of bronchodilators. As part of the quality assurance process, the pediatric pulmonologist performed *post hoc* inspection of the flow-volume curves to ensure their accuracy and reliability. For statistical analysis, the lung function parameter with the highest recorded measurement was selected from a series of three technically acceptable flow-volume curves (Miller et al., 2005). The FVC and FEV_1_ were measured, and their standardized z-scores were calculated by subtracting the predictive values and dividing the result by the standard deviation of the predictive values (Culver et al., 2017; Harris et al., 2018). The Global Lung Initiative (GLI) 2012 reference values were consistently applied to evaluate the spirometry data from our study population predominantly comprising individuals of non-Hispanic white ethnicity (Criswell et al., 2023; Quanjer et al., 2012).

### 2.5. Covariates

Structured questionnaires were used to gather comprehensive data on sociodemographic and lifestyle factors. These factors included maternal smoking status during pregnancy, classified as never smoker, former smoker, or current smoker. The highest level of education attained by the mother was also collected, categorized as less than 11^th^ grade or high school graduate or equivalent, junior college graduate or some college or technical school, college graduate, or any post-graduate schooling. Additionally, maternal age at enrollment was recorded as a continuous variable, measured in years. To determine maternal weight before pregnancy, measurements in kilograms were retrieved from prenatal medical records. Maternal height, measured in centimeters, was also obtained. These measurements were then utilized to calculate the maternal body mass index (BMI) as a continuous variable expressed in kg/m^2^. Information on the child’s sex, as documented at birth, was extracted from the delivery medical records, and categorized as male or female. During the study visit, the child’s age (measured in years), sex, and measured height (measured in centimeters) were assessed. These variables, along with maternal smoking status, were considered potential confounding factors based on previous studies (Farzan et al., 2016; Powers et al., 2019; Signes-Pastor et al., 2021; Stick et al., 1996).

### 2.6. Statistical analysis

Out of the total 419 children included in the study, 9 were excluded due to missing spirometry parameters. Additionally, 2 children were excluded due to spirometry parameter values outside the acceptable range. Furthermore, 73 children were excluded from the analysis as they lacked maternal urinary metal concentrations, and 19 children were excluded due to missing maternal smoking status. Our final complete case dataset contained 316 maternal-child pairs (Fig. S1) and was used for the main statistical analysis. Summary statistics were calculated for each variable: median (interquartile range) for continuous variables and *n* (%) of each level of categorical variables. The urinary metal concentrations, including the sum of urinary As species (ΣAs = inorganic As + monomethylarsonic acid (MMA) + dimethylarsinic acid (DMA)) were urine dilution (specific gravity) adjusted and log-transformed to address their positive skewness. The spirometry parameters had symmetric distributions, and their standardized z-scores were calculated as described previously (Culver et al., 2017; Harris et al., 2018). The FEV_1_/FVC ratio was also calculated to assess the presence and nature of clinical lung disorders (Powers et al., 2019).

Bayesian kernel machine regression (BKMR) was performed to flexibly investigate dose responses, interactions and joint effects between the urinary metal concentrations and the spirometry parameters of interest (Bobb et al., 2015, 2018). BKMR models were applied as $Y_{i}=h\left(\sum As_{i}+Cd_{i}+Co_{i}+Cu_{i}+Mo_{i}+Ni_{i}+Pb_{i}+Sb_{i}+Se_{i}+Sn_{i}+Zn_{i}\right)+\beta^{T}+Z_{i}+e_{i}$, where Y is the continuous outcome of interest (i.e., FVC and FEV_1_); h( ) is an exposure–response function that accommodates nonlinearity and interactions among metal mixture components natural log-transformed, centered and scaled (Fig. S2); Z are the selected covariates based on a prior studies (i.e., maternal smoking status, and child’s age, sex and height) (Farzan et al., 2016; Powers et al., 2019; Signes-Pastor et al., 2021; Stick et al., 1996) and β are the corresponding regression coefficients. All models included 10000 Markov chain Monte Carlo iterations using the Gaussian kernel, with 5000 used as burn-in. Additionally, the Weighted Quantile Sum Regression (WQSR) mixture approach was employed to construct a weighted index that captures the combined effect of all metals on FVC and FEV_1_. The WQSR model partitioned the dataset into 40% for training and 60% for validation. Parameter estimation was performed using 100 bootstrap samples. With the WQSR model, estimates of mixture effects and exposure importance indicators (referred to as weights) were derived by aggregating the metal exposures into an empirically weighted index (Carrico et al., 2015). Initial analyses indicated no substantial evidence of non-linear associations between maternal urinary metal concentrations and children’s FVC and FEV_1_. Hence, single, and multiple linear regression analyses were conducted to assess the association between metal exposures and the z-score spirometry parameters of the children. The models included FVC and FEV_1_ z-scores as dependent variables and log_2_-transformed concentrations of specific metals as independent variables. The models were adjusted for potential confounding factors, including maternal smoking status, children’s age, sex, and height. Associations were deemed statistically significant if they met a threshold of α = 0.05. The statistical analyses and graphics were performed using R software version 4.0 (R Core Team, 2021).

## 3. Results

The median maternal age of enrollment was 30.7 years, and about 91.8% were nonsmokers (*n* = 290). A total of 47.5% of children were boys (*n* = 150). The spirometry test was performed at children’s median age of 7.4 years with a median weight and height of 125.0 cm and 25.8 kg, respectively (Table 1).

Maternal urine concentrations (median) included ΣAs (3.45 μg/L), Cd (0.06 μg/L), Co (0.34 μg/L), Cu (5.23 μg/L), Mo (42.4 μg/L), Ni (0.86 μg/L), Pb (0.20 μg/L), Sb (0.02 μg/L), Se (29.2 μg/L), Sn (0.31 μg/L), and Zn (155 μg/L). A Spearman’s correlation coefficient ≥0.7 was observed for urinary concentration of i) ΣAs and Se, ii) Cd, Cu, Mo, and Se, and iii) Zn and Se (Fig. S3).

**Fig. 1.**
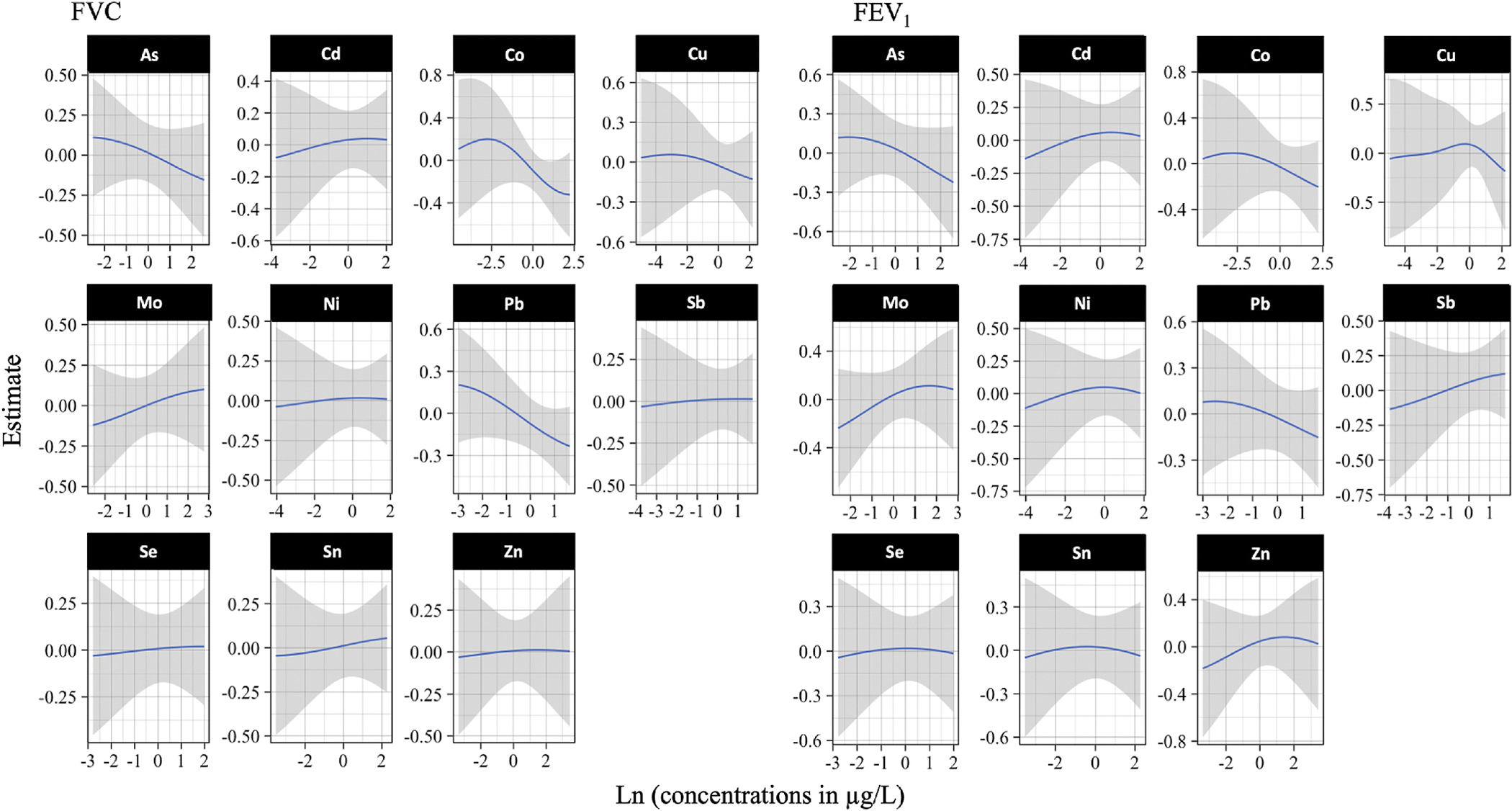
BKMR dose-response function for each metal of the mixture when the others are fixed at the median. *n* = 316. Ln-transformed maternal urinary metal concentrations specific gravity corrected as independent variables adjusted for maternal smoking status, children’s age, sex, and height. Notice that the scale of the y-axis varies to facilitate the visualization of the estimates in each plot.

The BKMR univariate exposure–response functions are shown in Fig. 1. They provide the associations between each metal, with the remaining included in the mixture fixed at the median, and FVC and FEV_1_ z-scores. An inverse association between ΣAs, Co, and Pb in the mixture and children’s FVC and FEV_1_ z-scores was observed and appeared to be linear. However, high variability was found between urinary Co and children’s FVC at low concentrations, suggesting a weak inverse U-shaped dose-response curve. The BKMR analyses did not reveal clear evidence of interaction between metals (Fig. S4). While the overall effect of the metal mixture showed an inverse trend with FVC and FEV_1_, this trend did not reach statistical significance (Fig. S5). The Posterior Inclusion Probability (PIP) values provided generally similar results (Fig. S6). Using WQSR, Mo had the highest positive weight for FVC (0.663) and FEV_1_ (0.778) and the highest negative to Co for FVC (0.507) and FEV_1_ (0.453) followed by As and Sb with similar weights (Fig. S7).

In multiple linear regression models, a doubling of maternal Co and Pb was associated with a decrease in FVC z-score of β = −0.09 (95% confidence interval (CI) −0.18 to −0.01) and β = −0.07 (95% CI −0.13 to 0.00), respectively. A decrease of β = −0.09 (95% CI −0.18 to 0.00) was also observed between Co concentrations and FEV 1 z-score. A doubling of maternal ΣAs was associated with a decreased of β = −0.09 (95% CI −0.20 to 0.01) in FVC z-score, and β = −0.08 (95% CI −0.20 to 0.04) in FEV_1_; however, the associations did not reach statistical significance. An increase of β = 0.11 (95% CI −0.04 to 0.26) and β = 0.15 (95% CI −0.02 to 0.32) was associated with urinary Mo concentrations and FVC and FEV_1_ z-scores, respectively, which was of borderline statistical significance (Table 2). The findings from the single linear regressions followed the trends reported in the multiple linear regression, except for Mo (Table S2). We did not observe any clear association between maternal urinary metal concentrations and children’s lung function z-scores for any of the other elements.

## 4. Discussion

This study focused on prenatal metal mixture exposure at levels found in a general population of the US (Lozano et al., 2022; Watson et al., 2020). Within the metal mixture, we observed consistent positive associations with prenatal Mo exposure and children’s lung function evaluated by spirometry, and negative associations with As, Co, Pb and Sb. The metal mixture exposure analyses indicated a linear dose-response with little evidence of interactions between metals.

Lung development starts approximately three weeks after fertilization and continues throughout early childhood. Critical stages of lung development occur during gestational weeks 16–25 (canalicular stage) and 24–38 (saccular stage) (Mullassery and Smith, 2015). In the canalicular stage, type I and II pneumocytes differentiate, and the alveolar capillary barrier forms. During the saccular stage, gas exchange areas increase (Joshi and Kotecha, 2007). However, exposure to toxic elements such as inorganic As, Cd, and Pb during pregnancy can cross the placental barrier and directly affect the fetus, leading to malformations and abnormal lung growth and development, often associated with impaired lung function (Hsieh et al., 2021). In utero exposure to inorganic As and Pb can also induce oxidative stress, damaging lung epithelial cells and triggering inflammatory cascades, which may lead to pulmonary diseases (Rosa et al., 2022; Signes-Pastor et al., 2021). Similarly, exposure to Co has been linked to lung diseases due to its ability to increase activated oxygen species (Linna et al., 2003). Combined exposure to Co and tungsten (W) may exacerbate the adverse effects in the occupational setting (Adams et al., 2017).

Several cross-sectional studies have investigated the association between metal exposures and lung function in childhood (Adams et al., 2017; Little et al., 2017; Madrigal et al., 2018, 2021; Powers et al., 2019). However, limited evidence exists on the effects of prenatal metal mixture exposure on pulmonary function in children. A study in Mexico City of 222 mother-child dyads reported inverse associations between prenatal blood Pb and lung function in 8–11-year-old children, similar to our findings in magnitude, but these associations did not reach statistical significance (Rosa et al., 2022). In our study, we observed a decrease in FVC but not in FEV_1_, indicating a restrictive lung pattern where the lungs cannot fully expand or fill with air but can still expel air effectively. Another study in Mexico reported that maternal blood Pb exposure (median 28.8 μg/L) was associated with higher wheeze in children aged 4–7 years, but they did not examine lung function (McRae et al., 2022).

No prior studies to our knowledge have examined prenatal Co exposure, either individually or as part of an exposure mixture in relation to children’s lung function. In a cross-sectional study conducted with healthy college students in China, a negative association was found between Co exposure (median = 0.76 μg/g, creatinine) and FVC and FEV_1_ (Zeng et al., 2022). Our study found Co concentrations in urine that were comparable to levels previously reported in studies that examined the general population (Ohashi et al., 2006). In our study, we observed a decrease in both FVC and FEV_1_ with higher Co concentrations, indicating a mixed pattern involving elements of both restriction and obstruction. However, it’s important to note that most of our study population remained within the healthy range, displaying no clinical signs of obstruction or restriction. Only approximately 2% of children exhibited clinical airflow obstruction (FEV_1_/FVC <0.70) or a restrictive pattern (FVC <80% predicted with FEV_1_/FVC ≥0.70) (Powers et al., 2019).

We found positive associations with Mo, a nutrient element, which is typically present in human urine at a concentration of about 50 μg/L, similar to the median value of 42.4 μg/L found in our study of pregnant women (Hadrup et al., 2022). Mo is a trace element with beneficial health effects, acting as a cofactor of sulfite oxidase, an enzyme that catalyzes the final step in oxidation of sulfur amino acids in mitochondria (Novotny and Peterson, 2018). Mo positively associated with FVC and FEV_1_ in Austrian metal plant workers (Ott et al., 2004), consistent with our findings in prenatal Mo exposure and children’s lung function at 7 years old. Experimental studies with mice and rats (Chan et al., 1998; Sobańska et al., 2020) assessed the association between Mo exposure and pulmonary toxicity, but further studies in humans are needed, focusing on vulnerable stages such as pregnancy.

We adjusted for potential confounding factors in our analysis, yet residual confounding remains a possibility. Urinary metal concentrations are a reliable biomarker of exposure, though excretion rates may vary across metals (Fort et al., 2014). Our study focuses on prenatal metal exposure and lacks data on childhood metal exposure, which could differ from maternal exposure. Additionally, our study primarily includes children without clinical signs of lung disorders. However, we still identified statistically significant associations between metal mixture exposure and the lung function parameters of interest. To reduce misclassification of inorganic As exposure, we estimated exposure by measuring urinary As species concentrations, including inorganic As, MMA, and DMA (Jones et al., 2016; Signes-Pastor et al., 2017). We employed common spirometry measures, such as total FVC and FEV_1_ after full inhalation, to evaluate lung function (David and Edwards, 2022). We used the non-parametric statistical approach BKMR to analyze mixture effects, which estimates the multivariable exposure response function in a flexible way and allows for non-linear and non-additive effects. The hierarchical variable selection approach helps address multicollinearity (Bobb et al., 2018). The WQSR method was also applied, estimating a joint effect of the entire mixture (Carrico et al., 2015). Nonetheless, our study sample size was modest and thus, we had limited statistical power, especially for detecting interaction effects.

The findings of this study suggest potentially detrimental impacts of prenatal exposure to certain toxic concentrations of metals such as Co and As, and possibly Pb and Sb, and potential benefits of higher Mo exposure within a metal mixture on children lung function. Large prospective studies on the effects of metal mixtures will help to understand the overall impacts of nutrient and toxic chronic metal exposures on lung development.

## Supplementary Material

Supplementary Material

## Figures and Tables

**Table 1:** Selected characteristics of the study mothers and children.

	Final sample
	
	*n* = 316

Maternal age of enrollment (years)	30.7 [28.3; 33.6]
Gestational age (weeks)	39.0 [38.4; 40.0]
Maternal pre-pregnancy BMI	24.3 [21.8; 28.3]
Maternal education:	
<11^th^ grade or high school graduate or equivalent	25 (7.9%)
Junior college graduate or some college or technical school	56 (17.7%)
College graduate	142 (44.9%)
Any postgraduate schooling	93 (29.4%)
Parity:	
0	128 (41.0%)
1	116 (37.2%)
>1	68 (21.8%)
Smoking pregnancy (yes/no)	26 (8.2%)/290 (91.8%)
Maternal status:	
Married	279 (88.3%)
Single	30 (9.5%)
Divorced	7 (2.2%)
Maternal urine concentrations (μg/L):	
∑As	3.45 [1.37; 5.92]
Cd	0.06 [0.03; 0.12]
Co	0.34 [0.16; 0.77]
Cu	5.23 [1.83; 9.80]
Mo	42.4 [20.2; 77.0]
Ni	0.86 [0.21; 2.19]
Pb	0.20 [0.07; 0.39]
Sb	0.02 [0.01; 0.05]
Se	29.2 [13.7; 50.6]
Sn	0.31 [0.13; 0.88]
Zn	155 [72.2; 333]
Children (boys/girls)	150 (47.5%)/166 (52.5%)
Spirometry age (years)	7.39 [7.06; 7.97]
Weight (kg)	25.8 [23.0; 29.4]
Height (cm)	125.0 [121.2; 129.3]
FVC	1.74 [1.53; 2.13]
FEV_1_	1.50 [1.39; 1.74]
FEV_1_/FVC	0.89 [0.85; 0.93]
FVC-zscore	0.41 [−0.30; 1.01]
FEV_1_-zscore	0.41 [−0.31; 1.05]

Continuous variables show median [first quartile; third quartile]. *n* = 316. Maternal BMI has 1 missing value. Parity has 4 missing values. ∑As = inorganic As + MMA + DMA.

**Table 2:** Multiple linear regression between metal concentrations in maternal urine samples during gestation and children FVC and FEV_1_.

FVC					FEV_1_			
								
metal	β	95% CI		*p*-value	β	95% CI		*p*-value

∑As	−0.09	−0.20	0.01	0.086	−0.08	−0.20	0.04	0.200
Cd	0.03	−0.08	0.14	0.607	0.01	−0.11	0.13	0.858
Co	**0.09**	**−0.18**	**−0.01**	**0.026**	**−0.09**	**−0.18**	**0.00**	**0.050**
Cu	−0.05	−0.13	0.04	0.288	−0.04	−0.14	0.05	0.373
Mo	0.11	−0.04	0.26	0.162	0.15	−0.02	0.32	0.086
Ni	0.03	−0.05	0.11	0.413	0.04	−0.05	0.13	0.396
Pb	**−0.07**	**−0.13**	**0.00**	**0.035**	−0.04	−0.11	0.03	0.235
Sb	0.03	−0.04	0.09	0.429	0.04	−0.03	0.11	0.231
Se	−0.04	−0.25	0.18	0.743	−0.11	−0.35	0.13	0.367
Sn	0.01	−0.05	0.06	0.840	−0.02	−0.09	0.04	0.508
Zn	0.04	−0.07	0.16	0.439	0.08	−0.05	0.20	0.228

*n* = 316. Multiple linear regression models with spirometry parameter standardized z-scores as dependent variables and log_2_-transformed maternal urinary metal concentrations adjusted for maternal smoking status, children’s age, sex, and height.

## Data Availability

Analytic data used in this study are included in the manuscript figures and tables and its Supplementary Information files

## Appendix A. Supplementary data

Supplementary data to this article can be found online at https://doi.org/10.1016/j.envres.2023.117234.
