# Supplementary material for: Prenatal exposure to metal mixtures and lung function in children from the New Hampshire birth cohort study
Source: Environ Res. Author manuscript; Available in PMC 2026 Feb 14. (PMC12906365; doi:10.1016/j.envres.2023.117234)

# Support Information

**Table S1**: Limit of detection (LOD) and imputed values in urine samples.

| Element | LOD (µg/L) - median | *n* (%) <LOD | *n* (%) imputed) |
| --- | --- | --- | --- |
| ∑As | 0.038 | 0 | 0 |
| Cd | 0.001 | 10 (3.2) | 0 |
| Co | 0.001 | 8 (2.5) | 0 |
| Cu | 0.150 | 30 (9.5) | 0 |
| Mo | 0.100 | 0 | 0 |
| Ni | 0.150 | 87 (27.5) | 0 |
| Pb | 0.006 | 39 (12.3) | 35 (11.1) |
| Sb | 0.002 | 102 (32.3) | 13 (4.1) |
| Se | 0.400 | 0 | 0 |
| Sn | 0.025 | 64 (20.3) | 8 (2.5) |
| Zn | 1.000 | 0 | 0 |

*n* = 316. The value of LOD/√2 was imputed only when the ICP-MS standard calibration curve provided zero or negative values (Lubin et al., 2004). The ∑As concentrations refer to the sum of inorganic arsenic, monomethylarsonic acid, and dimethylarsinic acid.

**Table S2**: Single linear regression between metal concentrations in maternal urine samples during gestation and children FVC and FEV_1_.

| FVC |  |  |  |  | FEV_1_ |  |  |  |
| --- | --- | --- | --- | --- | --- | --- | --- | --- |
| Metals | ß | 95% CI |  | *p*-value | ß | 95% CI |  | *p*-value |
| ∑As | **-0.10** | **-0.17** | **0.02** | **0.015** | -0.08 | -0.17 | 0.01 | 0.065 |
| Cd | -0.06 | -0.12 | 0.01 | 0.092 | -0.05 | -0.12 | 0.03 | 0.220 |
| Co | **-0.09** | **-0.15** | **-0.04** | **0.001** | **-0.08** | **-0.14** | **-0.02** | **0.010** |
| Cu | **-0.07** | **-0.12** | **-0.02** | **0.006** | -0.06 | -0.11 | 0.00 | 0.051 |
| Ni | -0.05 | -0.11 | 0.01 | 0.075 | -0.04 | -0.10 | 0.02 | 0.195 |
| Mo | -0.04 | -0.12 | 0.04 | 0.366 | -0.02 | -0.11 | 0.07 | 0.656 |
| Pb | **-0.07** | **-0.12** | **-0.03** | **0.003** | **-0.05** | **-0.10** | **0.00** | **0.068** |
| Sb | -0.03 | -0.08 | 0.02 | 0.178 | -0.02 | -0.07 | 0.04 | 0.605 |
| Se | -0.09 | -0.18 | 0.00 | 0.057 | -0.08 | -0.18 | 0.03 | 0.140 |
| Sn | -0.04 | -0.08 | 0.01 | 0.115 | -0.05 | -0.10 | 0.01 | 0.080 |
| Zn | -0.06 | -0.13 | 0.01 | 0.071 | -0.03 | -0.11 | 0.04 | 0.403 |

*n* = 316. Single linear regression models with spirometry parameters standardized z-scores as dependent variables and log_2_-transformed maternal urinary metal concentrations adjusted for maternal smoking status, children’s age, sex, and height.

**Figure S1**: Flowchart


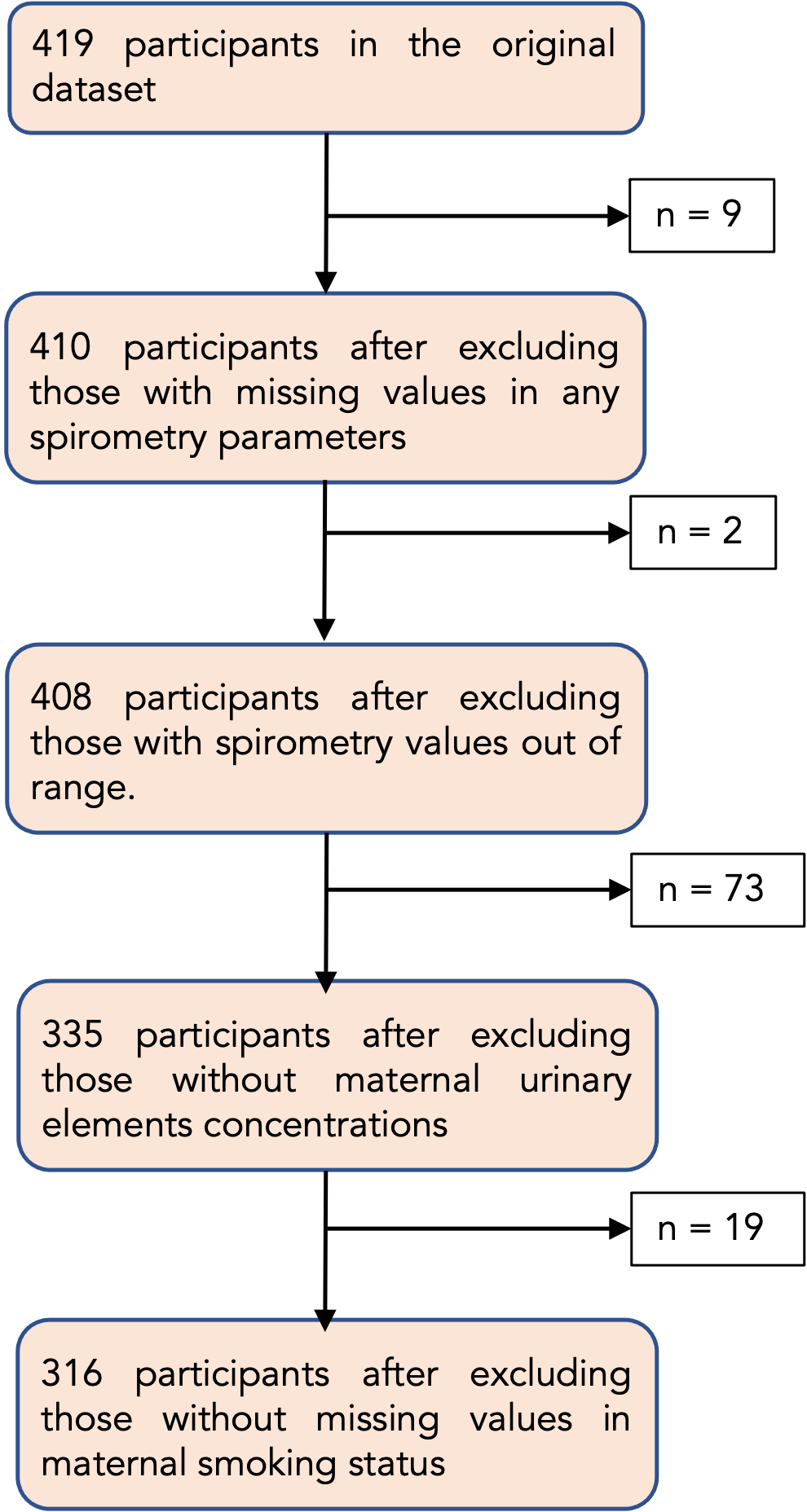


**Figure S2**: Density plot selected metals concentrations (*n* = 316)


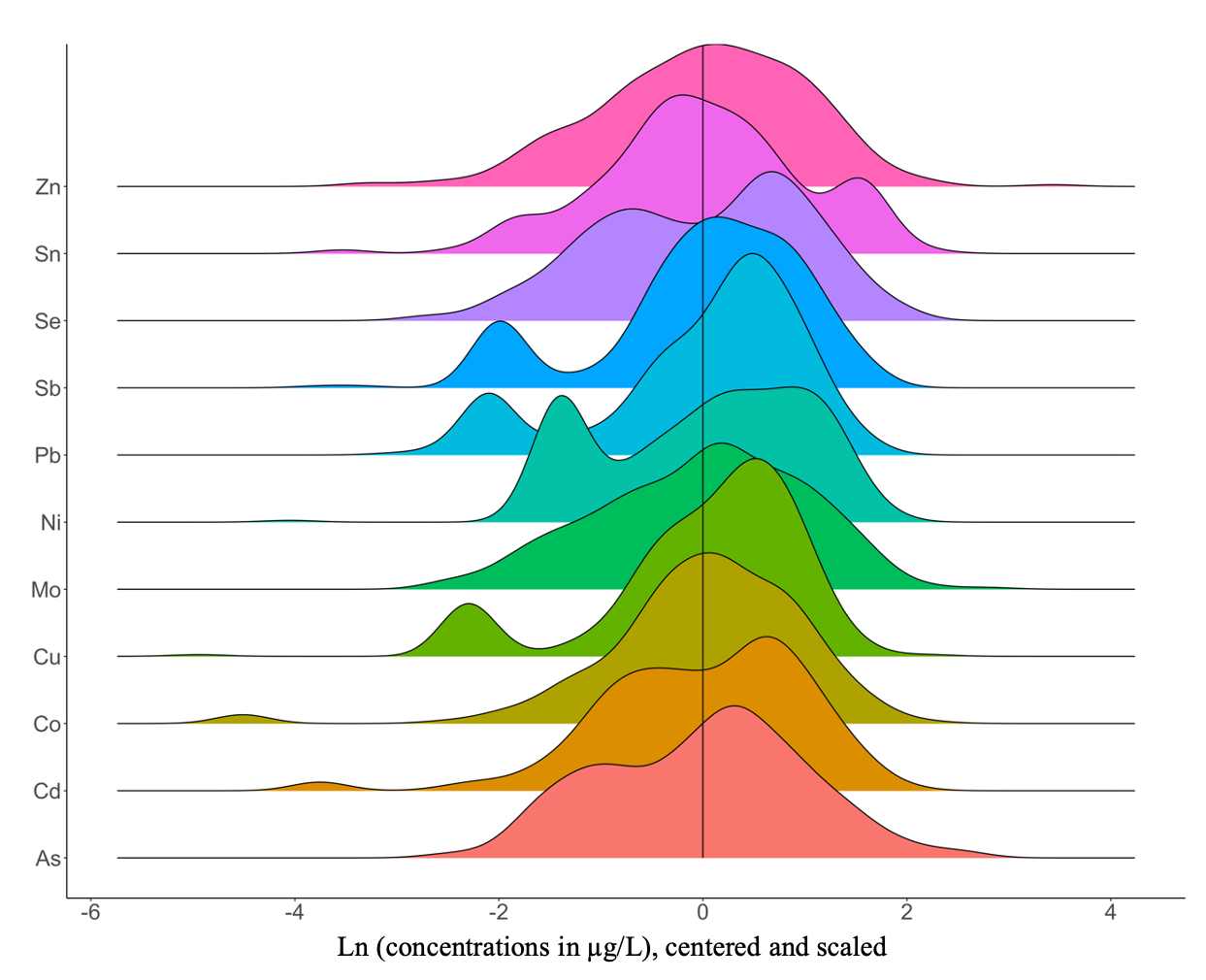


**Figure S3**: Spearman’s correlation matrix of maternal urinary metals concentrations (*n* = 316).


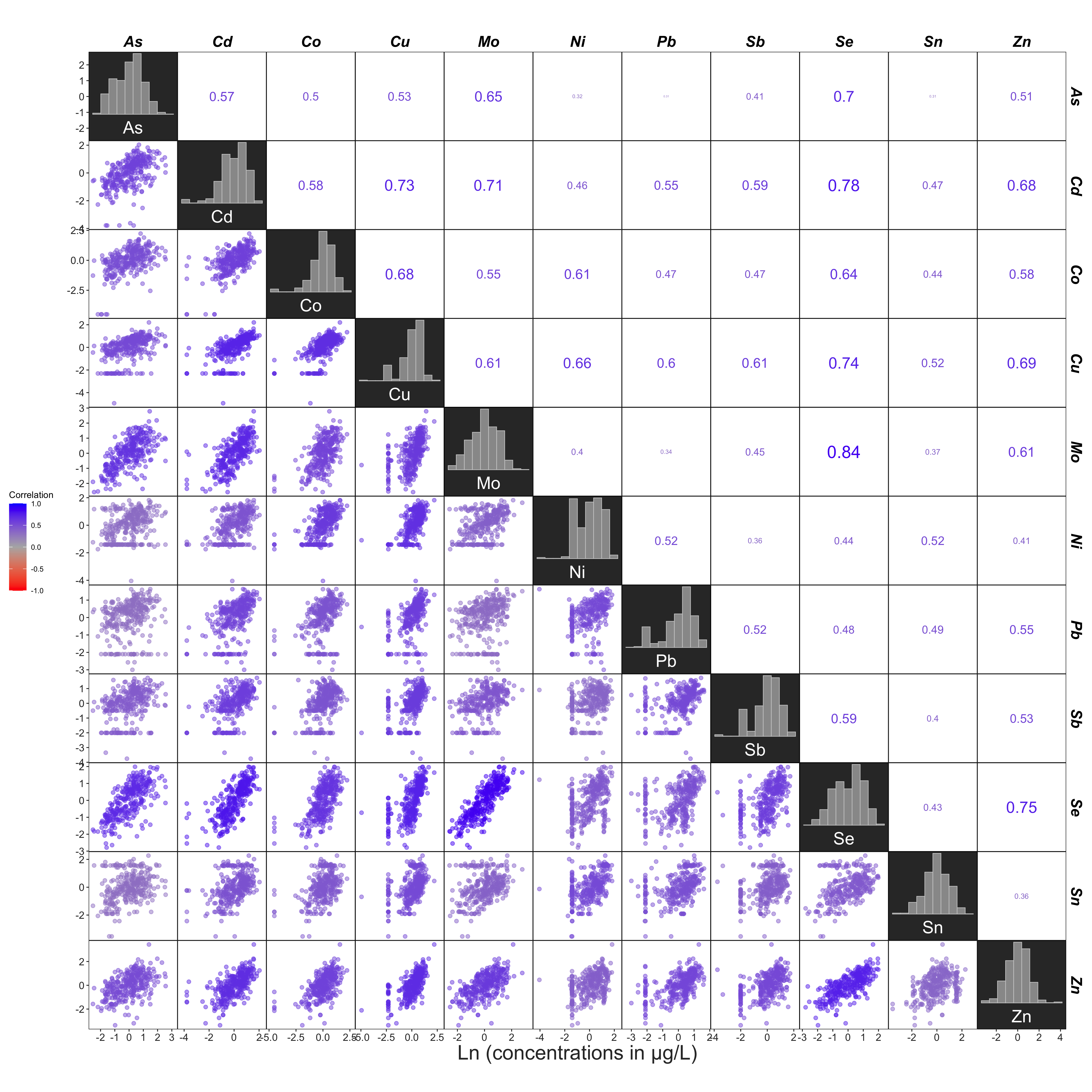


**Figure S4**: Single pollutant association within the mixture.


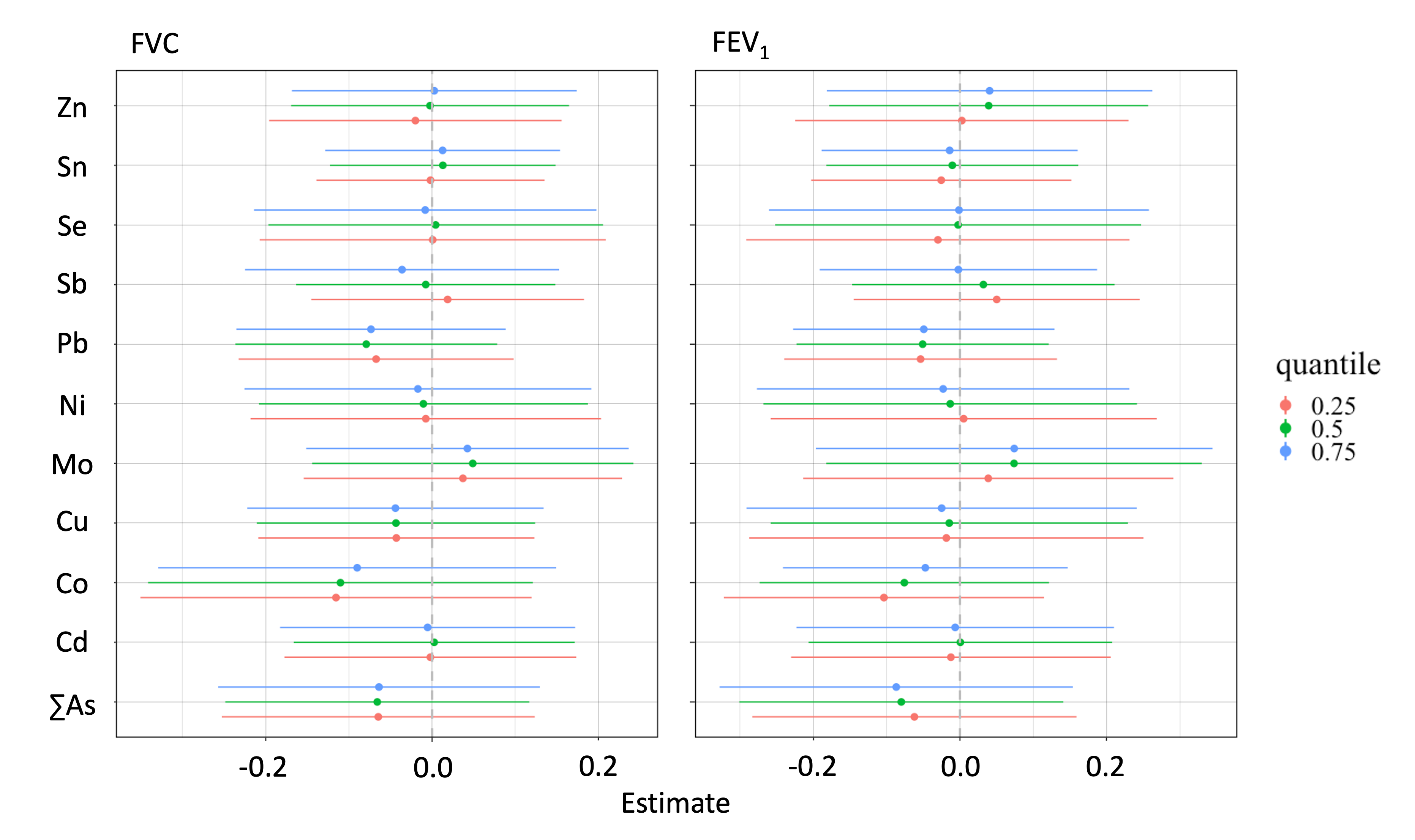


*n* = 316. Ln-transformed maternal urinary metal concentrations specific gravity corrected as independent variables adjusted for maternal smoking status, children’s age, sex, and heigh

**Figure S5**: BKMR cumulative mixture association with lung capacity.


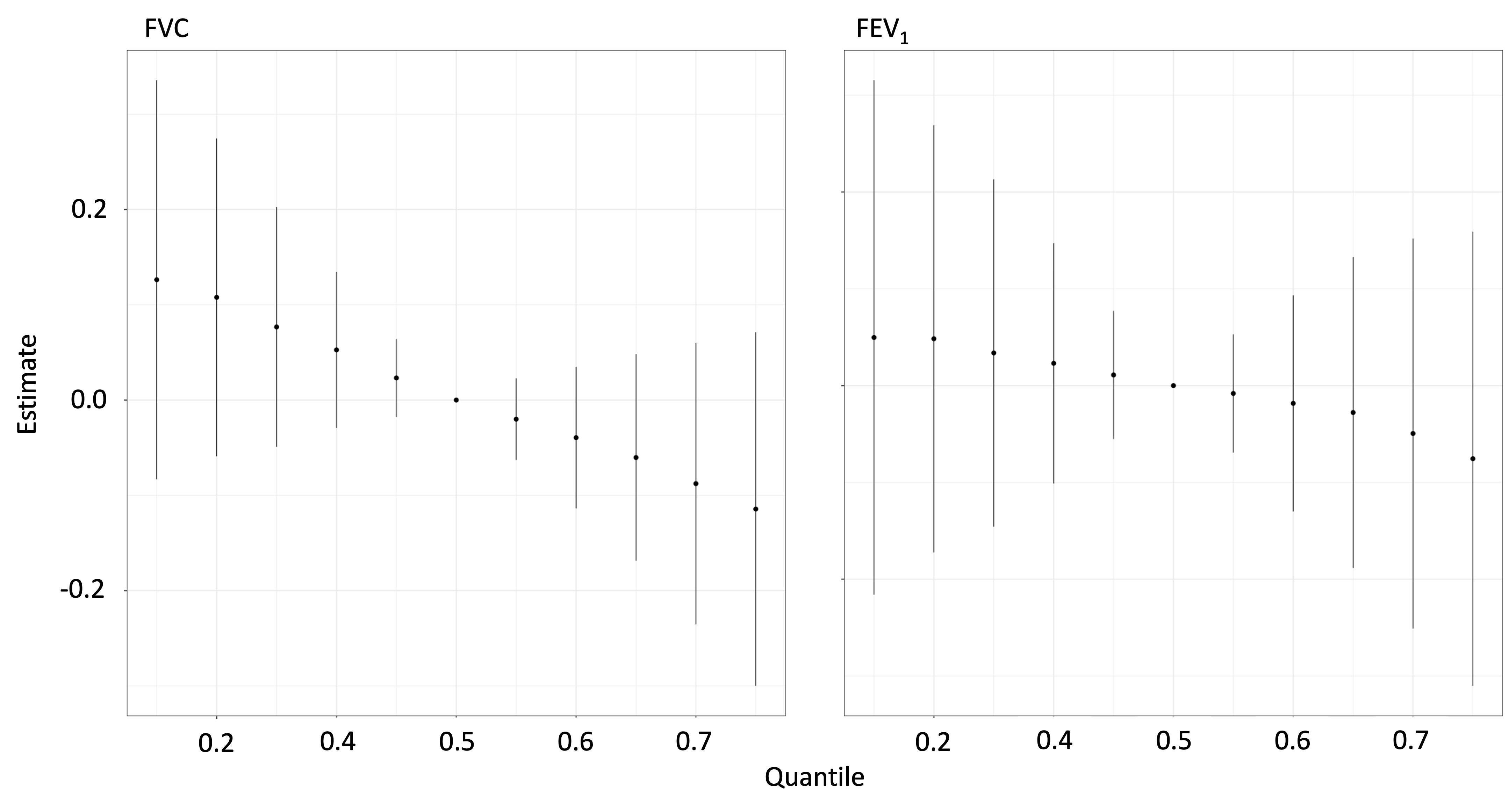


*n* = 316. Ln-transformed maternal urinary metal concentrations specific gravity corrected as independent variables adjusted for maternal smoking status, children’s age, sex, and heigh

**Figure S6**: Posterior Inclusion Probability (PIP).


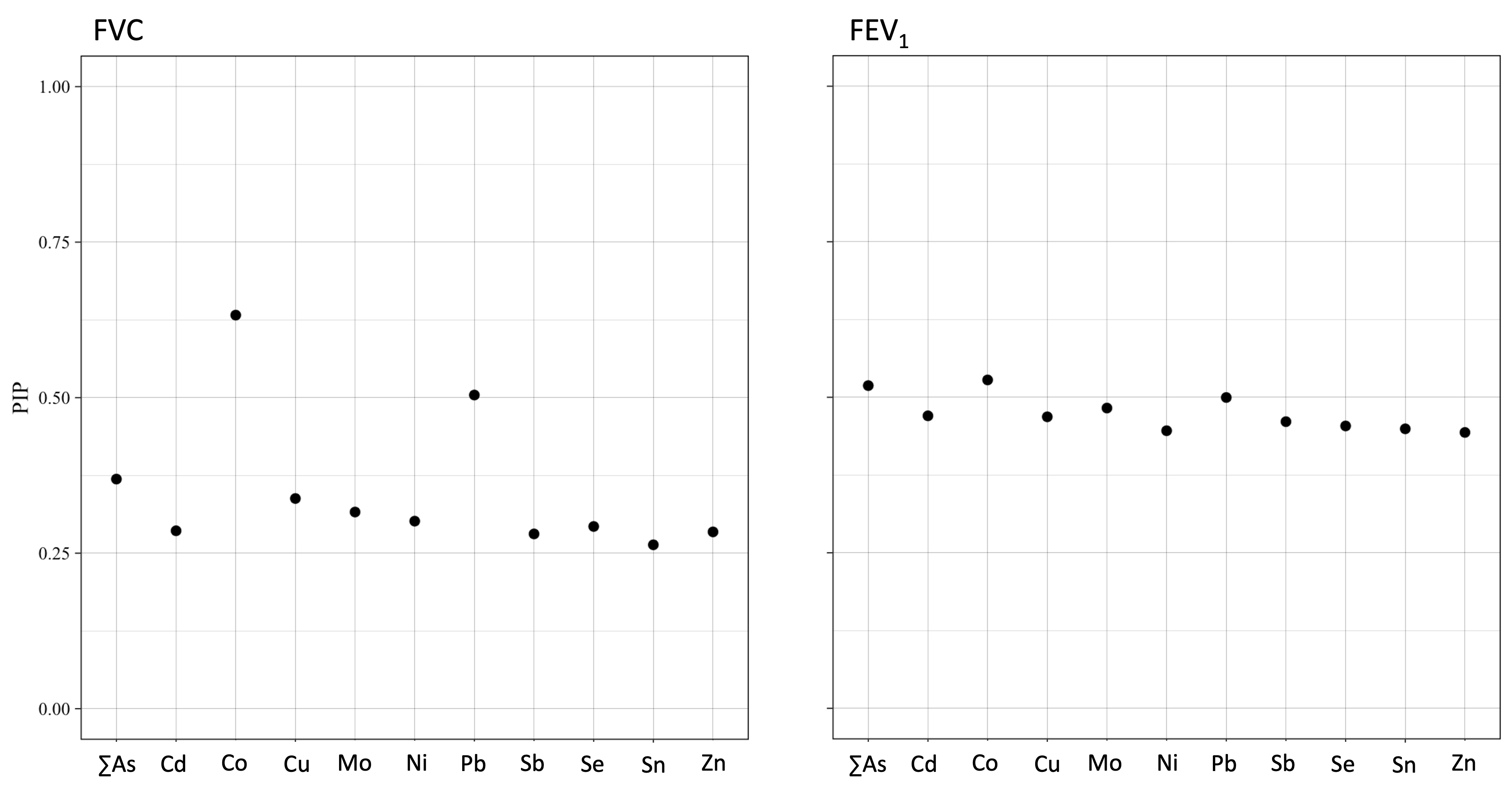


**Figure S7**: Weighted Quantile Sum Regression (WQSR) between maternal urine metal concentrations and FVC and FEV_1_.


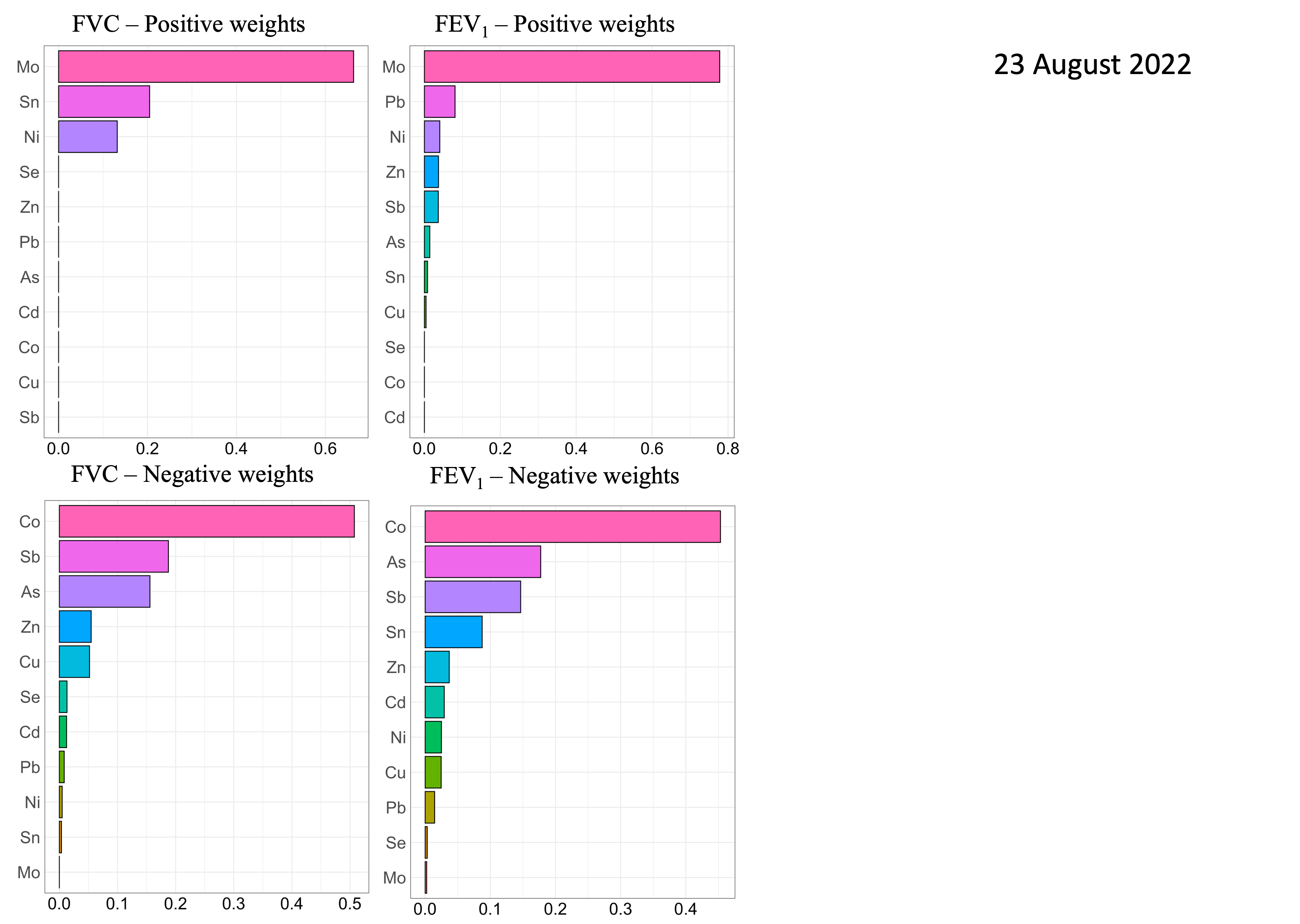

Supplement: Supplementary Material [file NIHMS2139062-supplement-Supplementary_Material.docx]
